# Combined Mutations of Canalicular Transporter Proteins Causing Low Phospholipid-Associated Cholelithiasis and Transient Neonatal Cholestasis in an Infant

**DOI:** 10.1097/PG9.0000000000000080

**Published:** 2021-04-22

**Authors:** Radhapyari Lourembam,, Rohan Malik,, Rishi Bolia

**Affiliations:** From the * Division of Pediatric Gastroenterology, Department of Pediatrics, All India Institute of Medical Sciences, Rishikesh, India; † Division of Pediatric Gastroenterology, Department of Pediatrics, All India Institute of Medical Sciences, New Delhi, India.

**Keywords:** BSEP, MDR3, progressive familial intrahepatic cholestasis

## Abstract

Low phospholipid-associated cholelithiasis syndrome is characterized by the development of cholelithiasis in early adulthood (<40 years of age) but is rarely diagnosed in childhood. It is associated with gene sequence variants in the ABCB4 gene encoding the multidrug resistance protein 3 which are mostly heterozygous. Transient neonatal cholestasis has been reported with heterozygous mutations in both ABCB4 and ABCB11 (Bile Salt Exporter Protein). We report a 3-month-old male with cholelithiasis and transient neonatal cholestasis in the setting of combined pathogenic heterozygous mutations in the genes ABCB4 and ABCB11. Initiation of ursodeoxycholic acid therapy led to a resolution of the cholestasis and gall stones. Our case highlights the complex nature of the genetics of cholestatic disorders.

## CASE REPORT

A 3-month-old male infant presented to our clinic with a history of jaundice since day 3 of life. The child had been born at term gestation (weighing 2.4 kg) to nonconsanguineous parents with an uneventful antenatal and perinatal period. He was third in birth order and his older siblings were well. The infant’s mother had undergone surgery for gall bladder calculi 2 years back at the age of 27 years. On examination, his weight and length were 3.7 kg (<−3 SD) and 66 cm (2 SD), respectively. He was active, alert and had icterus and a soft liver palpable 4 cm below the right costal margin. Laboratory investigations revealed conjugated hyperbilirubinemia (Total/direct bilirubin 6.94/4.67 mg/dl, respectively), elevated liver enzymes (aspartate aminotransferase107 U/L, alanine aminotransferase 64.9 U/L, alkaline phosphatase 919 U/L, and gamma glutamyl transpeptidase 83 U/L). Serum albumin 3.5 mg/dl and international normalized ratio 0.98 were normal. The complete blood count, kidney function test, and c-reactive protein levels were normal. Abdominal ultrasonography documented mild hepatomegaly and 2 gall stones (larger 7.5 mm and another 6.2 mm in the gall baldder and neck region). Work up for hemolytic causes was negative. Percutaneous liver biopsy showed features of marked intrahepatic and canalicular cholestasis with prominent bile ducts.

Genetic study was done which revealed digenic variations in ABCB4 and ABCB11 genes with the mutation c.1769G> A (p. Arg590Gln) in ABCB4 (heterozygous) and the mutation c.1772A>G (p. Asn591Ser) in ABCB11 (heterozygous). Treatment was started with ursodeoxycholic acid (UDCA) and fat soluble vitamins.

By the time of his first follow-up, 2 weeks after discharge, jaundice had decreased. At follow-up at 6 months of age, he is doing well and his jaundice has subsided completely. His liver enzymes have improved and a repeat ultrasonography abdomen showed resolution of the gall stones. He is still continued on UDCA and under our follow-up.

## DISCUSSION

Our patient had transient neonatal cholestasis and cholelithiasis in the setting of combined mutations in both ABCB4 and ABCB11. The mechanisms involved in the interplay of these mutations and their contribution to the clinical manifestations observed in our patient are uncertain and intriguing.

ABCB4 encodes for the canalicular transport proteins multidrug resistance protein 3 (MDR3), and its defects are implicated in a wide spectrum of cholestatic diseases of varying degrees of severity. This includes transient neonatal cholestasis (TNC), progressive familial intrahepatic cholestasis (PFIC) type 3, benign recurrent intrahepatic cholestasis (BRIC) type 3, intrahepatic cholestasis of pregnancy (ICP), and cirrhosis of adulthood (1). Heterozygous ABCB4 gene variants are also responsible for the so-called low phospholipid-associated cholelithiasis (LPAC) syndrome (2). In LPAC, a defect in ABCB4 function causes a decrease in the phospholipid content in the bile and an increase in cholesterol saturation increasing its lithogenicity. Our patient had a heterozygous mutation in the ABCB4 gene, which has been previously reported in a compound heterozygous state in a patient with PFIC3 (3).

ABCB11 (BSEP protein) transport bile salts in the hepatocyte and its mutations are linked to TNC, PFIC2, BRIC2, ICP, and drug-induced liver injury (4). The gene variant in our patient has been previously observed in a patient with ICP (5).

TNC is often multifactorial attributed to immaturity, perinatal distress, or hypoxia. However, in ~10% no obvious cause is found, and it occurs due to a genetic defect in the hepatobiliary transport system. Mutations in both ABCB4 and ABCB11 have been associated with TNC. Even though described more commonly with heterozygous mutations, it has recently been described in patients with biallelic ABCB11 variants too (4).

Cholelithiasis in infants is uncommon. Our patient did not have any other recognizable predisposing factor for cholelithiasis, and it is conceivable that the mutation in ABCB4 predisposed to it. The genetic variant seen in our patient was predicted to be damaging by SIFT and MutationTaster2. Such heterozygous ABCB4 variants have been associated with LPAC. The clinical criteria for LPAC are the presence of 2 of the 3 following criteria: age <40 years at onset of biliary symptoms, recurrence of biliary symptoms after cholecystectomy, and intrahepatic hyperechogenic foci detected by ultrasound (6). Our patient did not meet these criteria, but it is not surprising, as though these criteria are valid in young adults (<40 years), that it is seldom applicable in children (7,8). It is hypothesized that certain manifestation of LPAC such as intrahepatic lithiasis appears only on puberty even in carriers of pathogenic mutations in ABCB4. In our case, the presence of a family history of biliary lithiasis and response to UDCA therapy further supports the diagnosis of LPAC (6,8). To the best of our knowledge, there have been no reports of LPAC in infancy till date (8).

There are only a few reports of such combined mutations in the literature. A similar digenic variation has been reported earlier by Cardoso et al (9) in a 39-year-old Caucasian male with choledocholithiasis, hepatolithiasis, and persistent conjugated hyperbilirubinemia after retrieval of stones. The patient fulfilled the criteria for LPAC syndrome and responded to UDCA therapy. Our patient also showed a favorable response to UDCA leading to a resolution of cholestasis, cholelithiasis, and normalization of liver enzymes. Such a complex interaction has also been reported in a pair of sibling with a combined PFIC and LPAC phenotype. They had 2 heterozygous nonsynonymous variants of ABCB4 and were also heterozygous for a ABCB11 variant. These patients had recurrent symptomatic cholelithiasis and extensive biliary fibrosis that progressed eventually necessitating liver transplantation (10).

How our patient would behave in the future is a matter of speculation. It is likely that our child with this LPAC phenotype would continue to fare well on long-term UDCA therapy. However, there are some arguments for the contrary too. The normal gamma glutamyl transpeptidase at presentation also raises the question of a PFIC or BRIC type 2. There is some literature to suggest that a proportion of patients with PFIC type 2 may also respond, at least transiently to UDCA (11). Only further follow-up will help to understand the evolution.

Apart from the short follow-up duration, another limitation of our report is that due to financial constraints we could not carry out genetic analysis of the parents.

To conclude, we report an infant with TNC and LPAC with heterozygous mutations in ABCB4 and ABCB11 who responded to UDCA therapy. Apart from reporting LPAC in infancy, our report also highlights the complex nature of the genetics of cholestatic disorders.
